# Public Awareness of Ocular Chemical Injuries: A Cross-Sectional Study in Lithuania

**DOI:** 10.3390/clinpract15020035

**Published:** 2025-02-13

**Authors:** Justina Skruodyte, Martyna Sveikataite, Jurate Sveikatiene, Pranas Serpytis

**Affiliations:** 1 Faculty of Medicine, Vilnius University, LT-01131 Vilnius, Lithuania; 2 Eye Diseases Department, Republican Vilnius University Hospital, LT-04130 Vilnius, Lithuania; 3 Faculty of Medicine, Institute of Clinical Medicine, Vilnius University, LT-01131 Vilnius, Lithuania

**Keywords:** chemical eye burns, ocular trauma, eye injuries, emergency eye care

## Abstract

*Background:* Ophthalmic emergencies are acute conditions that progress rapidly, posing a significant threat to a patient’s vision and requiring urgent intervention to prevent permanent visual impairment. This study aimed to assess the general awareness of ocular chemical burns and the adequacy of the immediate response measures while also seeking to improve the understanding of these injuries and contribute to promoting a healthier society. *Methods:* A comprehensive literature review was performed, and the most frequently reported questions were incorporated into the original survey. An anonymous questionnaire, available both online and in print, was developed to conduct a cross-sectional study to assess the general knowledge of the causes, symptoms, and appropriate first aid measures to be applied in cases of chemical ocular trauma. *Results:* Between April and May 2024, 175 individuals completed the questionnaire and were included in the study. More than half (54%) of the tested population demonstrated a poor level of knowledge about chemical ocular injuries, and only 30.9% of the respondents correctly indicated the need for imminent treatment. Twenty percent of the respondents could not identify whether alkalis or acids are more hazardous. Additionally, 5.7% of the respondents falsely considered consultation with an emergency department a priority before thorough irrigation. Most of the respondents (60.6%) incorrectly indicated that the appropriate first aid measures and treatment depend on the substance involved, and 68.1% of the respondents stated that treatment in the emergency department should be delayed, allowing for anamnesis collection and thorough examination. *Conclusions:* Ocular chemical injuries can substantially impact an individual’s quality of life. The present study found that the public knowledge concerning ocular chemical trauma, and the necessary immediate treatment is insufficient. Public education is vital, as delaying prompt and thorough irrigation at the chemical injury site may result in irreversible complications.

## 1. Introduction

Ophthalmic emergencies are acute conditions that progress rapidly and threaten a patient’s vision, necessitating prompt treatment to prevent permanent vision loss. The morbidity associated with ophthalmic trauma varies significantly, ranging from minor, inconsequential conditions to those with potential vision-threatening consequences [1]. A study conducted at the Emergency Department of Zhongshan Ophthalmic Center revealed that up to 44.1% of patients presented with ocular trauma. However, a substantial number of visits were due to minor ailments that mimicked urgent conditions [2]. Ocular chemical burns have been diagnosed in up to 22.1% of patients with ocular injuries [3,4,5]. They represent the second-most prevalent type of occupational eye injury (12.68%), following injuries caused by foreign bodies. A higher prevalence is observed among young male patients, particularly those employed in the industrial sector [6,7]. Another vulnerable group consists of female-dominated service sector workers, particularly those in cleaning and kitchen roles, experiencing a high prevalence of chemical splashing and spraying [8]. The severity of ocular injuries is influenced by various factors, including the nature of the offending agent, the duration of exposure, the surface area affected, and the specific ocular tissues involved. In the case of chemical burns, the persistence and exacerbation of tissue damage depend on the continued contact of the chemical with the eye [9,10]. Any liquid or solid material containing acidic or alkaline components can cause ocular injuries [3]. Alkali substances, accounting for 60% of ocular chemical burns due to their extensive industrial and domestic use, are more frequently implicated in scleral necrosis than acids because their hydroxyl ions induce the saponification of fatty acids in the cell membrane, allowing the alkali to penetrate deeper into the corneal stroma, leading to damage affecting the cornea and the structures within the anterior chamber. Conversely, acid injuries cause protein denaturation and tissue coagulation, which usually limit the depth of acid penetration [11,12,13,14].

Chemical burns must be addressed without delay at the site of injury. A postponement in care allows the chemical agent to penetrate deeper, leading to extensive damage and involvement of the extraocular and intraocular structures [5]. The affected eye(s) must be thoroughly irrigated with a non-caustic fluid, if available, at the location of the injury and during transfer to the hospital. This must be continued until the ocular surface is stabilized and the surface pH returns to the normal range of 7.0–7.2 to prevent complications such as scleral necrosis, corneal melting, and corneal neovascularization [3,15,16]. The rapid initiation of irrigation is more important than determining the substance’s chemical composition or waiting for particular irrigation fluids [3]. Tap water is the most readily accessible and commonly used rinsing solution, meeting most industrialized nations’ essential purity, sterility, and neutral pH requirements. Alternative rinsing solutions with higher osmolality have been proposed to more effectively stabilize the physiological pH through improved buffering capacity. However, there is currently no strong evidence to suggest a significant clinical advantage [17]. The frequent application of preservative-free artificial tears and biological solutions is beneficial for removing residual offending agents, controlling inflammation, preventing infection, and promoting the epithelialization and vascularization of the affected area [3,18]. The economic impact of ocular chemical trauma is substantial and escalates significantly with increasing injury severity, particularly in cases of bilateral injury. Chemical burns to the eye and adnexa represent a significant issue in the United States, accounting for approximately 36.000 emergency department visits annually and incurring USD 26.6 million in associated emergency department charges [6]. Another study in the United States estimated that direct treatment costs for ocular chemical injuries from 2010 to 2013 totaled USD 106.7 million, though this figure may be underestimated [6]. The median individual treatment expenses among the Chinese population were approximately USD 5.900 per patient [19]. The efficacy of eye personal protective equipment in preventing severe ocular injuries is well documented [20,21]. Despite the availability of information and public health initiatives, adopting simple and effective interventions, such as using protective eyewear and the immediate irrigation of the eyes, continues to pose a significant challenge. Protective eyewear mandated by the workplace proved to virtually eliminate the occurrence of occupational injuries in the Norwegian population [22]. The provision of general recommendations and the availability of protective eyewear is more critical than ever, particularly given the rise in self-initiated home improvement projects and the corresponding increase in injuries occurring in domestic settings following the COVID-19 pandemic [23,24]. A recent study in Saudi Arabia indicated that 8.4% of the respondents incorrectly believed that acidic eye injuries should be treated with alkaline solutions and vice versa, which is highly dangerous [25]. The inadequate initial management of chemical ocular burns may result in prolonged recovery periods, suboptimal clinical outcomes, and irreversible damage to the eyes and adnexa. After the injury, considerable adverse effects on visual function, the health-related quality of life, and psychological well-being have been documented. Individuals affected by the condition display elevated rates of anxiety, depression, and psychological distress in comparison to the general population [12,14]. This study sought to assess the overall level of awareness and identify the common misconceptions regarding ocular chemical burns to enhance the understanding of these injuries and contribute to advancing a healthier society. It further examined the variations in awareness across diverse demographic groups, including age, gender, educational attainment, and occupational categories. Currently, there are no officially implemented eye injury prevention programs in Lithuania. Therefore, this study aimed to provide foundational data to guide the development of future educational initiatives and to improve the public knowledge and emergency responses to chemical ocular injuries.

## 2. Methods

A comprehensive literature review was conducted, and the most frequently reported questions were incorporated into the original survey [25,26,27,28,29]. An anonymous questionnaire comprised questions about the respondent’s age, sex, and educational background. A pilot survey was then conducted with a limited sample of participants (*n* = 20), which was not included in the results. All the participants were assured of the anonymity of data collection to mitigate the potential bias associated with self-reported data. The participants’ knowledge of chemical ocular injuries was evaluated through six questions focused on general information about alkaline and acidic substances and the immediate treatment protocols for ocular burns. Each question was scored with 1 for a correct response and 0 for an incorrect or “don’t know” response. Therefore, the total score for all six questions ranged from 0 to 6 points. The participants’ overall knowledge was classified according to a modified version of Bloom’s cut-off criteria: “good” if the score was between 80% and 100% (5–6 points), “moderate” if the score ranged from 50% to 79% (3–4 points), and “poor” if the score was less than 50% (<3 points). The ongoing study assesses the general understanding of the causes, symptoms, and first aid measures that can be employed in the event of chemical ocular trauma in Lithuania. Data for this cross-sectional study were extracted from April to May 2024. The questionnaire was made available in two formats: online and in a physical form. The digital form was hosted in Google Forms, and data were gathered using snowball sampling via online social media platforms. Consent was indicated after the respondents clicked the “Go to Survey” button. The physical format of the questionnaire was distributed at The Republican Vilnius University Hospital for employees and Vilnius University medical students. In this instance, consent was indicated by the respondents’ agreement to complete the questionnaire. A total of 175 individuals completed the questionnaire, comprising 63 who completed the physical form and 112 who completed the online survey. According to the laws of the Republic of Lithuania, anonymous surveys do not require approval from a biomedical ethics committee. Respondents consent to participate in the study by completing the questionnaire after reading the introductory section, which provides information about the research being conducted, the study’s objectives, and how and where the results will be made available (Law of the Republic of Lithuania on Biomedical Research Ethics VIII-1679). The collected datasets were processed and analyzed using IBM SPSS 27.0 software. Frequency tables were used to portray the overall distribution of data. Qualitative variables were described using numbers and percentages. Cross-tabulations were performed to determine the percentage distributions of the nominal variables, and the data were analyzed according to the χ^2^ criterion, Fisher’s criterion (for 2 × 2 cross-tabulations), and the Z-test with Bonferroni correction. Statistical significance was set at *p* < 0.05.

## 3. Results

The age range of the participants was 18 to 75 years old, with a mean age of 36.44 years old. The participants were divided into three age-based groups. The majority of the respondents were female (78.9%, 138). A total of 94 participants (53.7%) had obtained a higher university education. A total of 86 participants (49.1%) indicated that they or someone they knew had previously sustained an ocular chemical injury. Moreover, 23 participants (13.1%) stated that they had previously treated a patient with this condition (Table 1).

The respondents’ knowledge of ocular chemical injury and first aid application is summarized in Table 2. Most of the respondents chose the correct answer and indicated that ocular chemical injuries from contact with alkali substances are the most prevalent and pose a greater risk than those caused by acidic substances (53.1%). A total of 35 respondents (20%) could not identify which substance, alkalis or acids, is more hazardous. The majority, 92% of the respondents, demonstrated sufficient knowledge regarding treatment strategies and indicated that immediate irrigation with water is crucial following a chemical injury. Nevertheless, 5.7% (10) of the respondents deemed consultation with an emergency department to be a priority.

It was established that participants within the 26–40 age bracket (15.4%) and those above the age of 41 (22.6%) had considerably more experience in treating patients with chemical eye burns in comparison to those below the age of 25 (1.6%) (*p* = 0.002). (Table 3). Most of the respondents (60.6%, 106) indicated that the appropriate first aid measures and treatment for a chemical burn depend on the specific substance involved. Additionally, 30.3% (53) of the participants emphasized the importance of a thorough medical history before initiating emergency department treatment. A total of 54 respondents (30.9%) selected the correct answer, indicating the need for urgent treatment. The proper response was significantly more common among those respondents who were still studying (*p* = 0.017).

**Figure 1:**
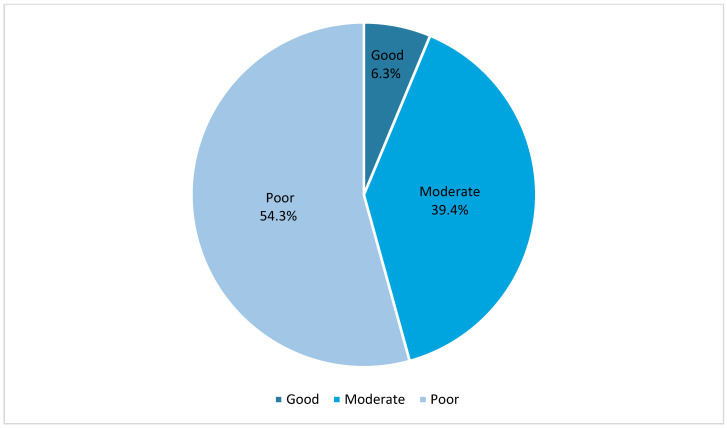
Representation of participants’ knowledge of chemical ocular injuries.

A statistically significant correlation was observed between those respondents with higher education levels and the belief that first aid measures depend on the substance causing the burn (*p* = 0.008). No statistically significant differences in answers were observed between the sexes. The analysis revealed no statistically significant differences between the various participant groups, categorized by their level of education or occupational background, in the immediate actions required after ocular chemical trauma or in the initial first aid measures administered by physicians in the emergency department. Figure 1 illustrates the percentage distribution of participants according to their knowledge about ocular chemical injury. The prevalence of good, moderate, and poor knowledge levels among the respondents was 11 (6.3%), 69 (39.4%), and 95 (54.3%), respectively.

The level of the participants’ knowledge about ocular chemical injuries according to their demographic data and personal experience is presented in Table 4. No statistically significant differences were observed between the age groups, two sexes, occupational statuses, or personal experience of ocular chemical injury. A statistically significant difference in the level of knowledge was noted between the respondents who already had experience treating ocular chemical injury and those who did not (*p* > 0.01).

## 4. Discussion

It can be argued that vision is the most important human sense. The loss of vision resulting from chemical injury can significantly impact an individual’s quality of life [30]. This study aimed to evaluate the awareness and knowledge of the immediate corrective action in cases of chemical eye injury among the general public and persons involved in medicine to reduce the incidence of ocular injuries and related complications. In this study, the highest incidence of ocular chemical burns was reported by individuals aged 41 years and older. No significant difference between the two sexes was observed. A total of 18.9% of the participants in the survey had a history of ocular chemical injury before the study. This figure is situated between the results of two studies conducted in Saudi Arabia, where 47.6% and 8.1% of the participants, respectively, had a previous history of ocular chemical trauma [25,26]. It is probable that the global incidence of ocular chemical injuries is underreported in the current literature because individuals with acute injuries may not always seek medical attention due to their limited access to healthcare. Instead, they may turn to community resources, such as family members, local pharmacies, or even chemical suppliers [14]. The majority of the published literature indicates that young working adult males are at the highest risk of sustaining ocular chemical injuries [8,14,31]. The severity of an ocular injury is contingent upon several factors, including the specific chemical agent involved, the volume and pH (alkaline or acidic) of the solution, and the duration of exposure. Alkaline solutions penetrate tissues with greater rapidity than acidic, thereby causing greater harm to intraocular structures, which can result in rapid and irreversible damage. Most of the participants in this study believed that alkali injuries were more dangerous and more prevalent in the domestic environment. A comparable outcome was observed in an Indian study [29]. Conjunctival congestion, often referred to as “red-eye syndrome”, has historically been regarded as an indicator of ocular trauma. However, the severity of the injury is proportional to the extent of limbal and scleral ischemia, which presents as a pale conjunctiva due to diminished blood flow [32]. The management of ocular chemical injuries can be generally categorized into acute and late-stage care. Acute management focuses on removing the harmful agent, controlling inflammation, and promoting the healing of the ocular surface, while late-stage management addresses the treatment of complications and visual rehabilitation. In the event of a chemical eye injury, the initial response should be prompt irrigation and dilution of the chemical with water to prevent irreversible damage. Imminent treatment does not depend on a causative agent and should not be delayed by determining it. The research findings in both experimental and clinical settings indicate that the early intervention employed significantly influences the process of epithelial regeneration and the clearance of corneal opacity [33]. In our study, 92% of the respondents indicated that rinsing the eyes with water should be the primary corrective measure. A similar outcome was observed in a study from Saudi Arabia, in which 78.5% of the respondents indicated that washing their eyes with water should be the first course of action. In the same study, however, 8.4% of the respondents suggested that acidic eye injuries should be rinsed with an alkaline solution, and a similar proportion believed that alkaline eye injuries should be treated with an acidic solution, which is highly dangerous [25]. Likewise, 60.6% of the participants in this study exhibited a misconception about the correct first aid procedures for chemical injuries, believing that these measures differ based on the substance in question. Conversely, 5.7% of the respondents indicated that the initial action should be to seek emergency medical assistance. A third of the participants believed that emergency department treatment should be initiated without delay. Nevertheless, a comparable proportion of the respondents falsely suggested that a comprehensive medical history should be obtained before the commencement of treatment. The appropriate management of alkali and acid burns, aimed at promoting epithelial integrity, enhancing stromal stability, minimizing excessive inflammation, and preventing complications, is initiated immediately at the incident scene through extensive irrigation, preferably with sterile irrigating fluid. It is not advisable to delay irrigation to await a more optimal solution. Given its accessibility and prevalence, tap water is the rinsing solution of choice in most industrialized countries. It is suitable for many applications due to its purity, sterility, and neutral pH [3,17]. This study revealed a limited level of knowledge regarding ocular chemical injuries among the respondents. Similar findings were observed in a study conducted in Saudi Arabia among medical students, where 73% of the participants demonstrated inadequate knowledge about ocular trauma. In contrast, a recent study in Saudi Arabia reported notably different results, with 56.62% of the participants exhibiting a strong understanding of ocular chemical injuries and the first aid measures [27]. These divergent findings may be attributed to the fact that a significant proportion (73.6%) of the respondents in the latter study had attained a higher education [26]. Implementing effective prevention strategies and providing thorough patient counseling are essential to mitigate the risk of eye injuries. A study conducted in the United Arab Emirates revealed that 85% of small-scale industrial workers participated in activities that posed a risk of eye injury and were aware of the associated hazards. However, none of them consistently wore safety goggles [34]. Research in Canada found that about 70% of individuals who suffered occupational eye injuries had not used eye protection [35]. Appropriate protective eyewear, such as safety glasses, face shields, and helmets, can prevent approximately 90% of eye injuries. Employers should enforce safety regulations, provide suitable protective equipment, and ensure that employees receive proper training in eye safety protocols, particularly in high-risk industries such as construction and manufacturing [36]. The primary limitation of this study lies in its small sample size and localized nature, which may limit the generalizability of the findings, particularly outside of Lithuania. Additionally, the self-reported design of the study introduces the potential for recall bias. Finally, the sample was predominantly composed of female participants, whereas the current literature indicates that most individuals experiencing ocular chemical burns are male.

## 5. Conclusions

An ocular chemical injury leading to vision impairment is a serious condition that can significantly diminish an individual’s quality of life, often resulting in job loss and an increased reliance on others. The current study revealed that the public awareness regarding ocular chemical injuries and the essential immediate treatment is lacking. Delaying prompt and thorough irrigation of the chemical injury site may result in irreversible complications. However, the public understanding of the first aid for chemical eye injuries can be enhanced through regular health education initiatives and increased efforts by healthcare professionals to communicate the key protective steps to take in the event of such an injury. We recommend expanding the study’s findings through further research and the implementation of targeted interventions, including public awareness campaigns on ocular chemical injuries and prompt corrective actions, distributing informational materials in workplaces and schools, and promoting the use of protective eyewear. Additionally, integrating simulation sessions and clinical case studies can enhance preparedness and equip individuals with the essential first-aid skills to effectively preserve vision. Collaborations with health organizations and healthcare policymakers can help drive policy changes and ensure the compliance with safety standards.

## Figures and Tables

**Table 1:** Demographics.

Respondent Characteristic		Number (*N* = 175)	Percentage (%)
Age (years)	<25	61	34.9
	25–40	52	29.7
	≥41	62	35.4

Sex	Female	138	78.9
	Male	37	21.1

Education	Secondary	7	4.0
	Currently studying	15	8.6
	Vocational	27	15.4
	Higher non-university education	27	15.4
	University education	94	53.7
	Other	5	2.9

Have you ever experienced an ocular chemical injury?	Yes (Me)	33	18.9
	Yes (Someone I know)	53	30.3
	No	89	50.9

Have you ever treated an ocular chemical injury?	Yes	23	13.1
	No	152	86.9

*N*, number of participants. %, percentage of participants.

**Table 2:** Response distribution across survey items.

	Responses	Number (*N* = 175)	Percentage (%)
Alkaline burns are more dangerous than acid burns.	Yes	93	53.1
	No	30	17.1
	I don’t know	52	29.7

What are the most prevalent substances that result in chemical burns within the domestic environment?	Acids	43	24.6
	Alkalis	58	33.1
	Acids and alkalis are equally common	39	22.3
	I don’t know	35	20.0

Following a chemical eye burn, what course of action should be initiated first?	Abundant rinsing with water	161	92.0
	Rinse with a small amount of water	3	1.7
	Go to the emergency department	10	5.7
	Apply artificial tear drops	1	0.6

What are the initial steps that medical personnel should take upon the arrival of a patient who has sustained a chemical injury to the eye?	Taking a medical history	53	30.3
	Thorough injury assessment	26	14.9
	Initial eye examination	40	22.9
	Immediate treatment	54	30.9
	I don’t know	2	1.1

Does first aid treatment differ depending on the origin of the substance causing the burn?	Yes	106	60.6
	No	52	29.7
	I don’t know	17	9.7

What fluid can be used for washing after a chemical eye burn in a medical facility	0.9% NaCl solution	94	53.7
	Tap water	44	25.1
	Any non-harmful liquid	25	14.3
	Other	12	6.9

*N*, number of participants. %, percentage of participants.

**Table 3:** Experience in treating ocular chemical injuries by age group.

			Age Groups (Years)		
			<25	25–40	≥41
Have you ever treated an ocular chemical injury?	Yes	Count	1_a_	8_b_	14_b_
		% within the age group	1.6%	15.4%	22.6%
	No	Count	60_a_	44_b_	48_b_
		% within the age group	98.4%	84.6%	77.4%
	Total	Count	61	52	62
		% within the age group	100%	100%	100%

Each subscript letter denotes a subset of age categories whose column proportions do not differ significantly from each other at the 0.05 level. Χ^2^ = 12.138; df = 2; *p* = 0.002.

**Table 4:** Representation of participants’ knowledge of chemical ocular injuries according to their demographic data and personal experience.

		Level of Knowledge % (*N*)		
		Good	Moderate	Poor
Age	≤25 years old	8.3 (5)	45.0 (27)	46.7 (28)
	>25 years old	5.2 (6)	36.5 (42)	58.3 (67)
Χ^2^ = 2.304 df = 2 *p* = 0.316				
Sex	Female	6.5 (9)	38.4 (53)	55.1 (76)
	Male	5.4 (11)	43.2 (16)	51.4 (19)
Χ^2^ = 0.305 df = 2 *p* = 0.858				
Education	Higher university education	5.3 (5)	36.2 (34)	58.5 (55)
	Other	7.4 (6)	43.2 (35)	49.4 (40)
Χ^2^ = 1.516 df = 2 *p* = 0.468				
Current occupation	Student	10 (4)	47.5 (19)	42.5 (17)
	Other	5.2 (7)	36.6 (49)	58.2 (78)
Χ^2^ = 4.997 df = 2 *p* = 0.288				
Experience of ocular chemical injury	Yes	7.0 (6)	38.4 (33)	54.7 (47)
	No	5.6 (5)	40.4 (36)	53.9 (48)
Χ^2^ = 0.180 df = 2 *p* = 0.914				
Experience in treating an ocular chemical injury	Yes	26.1 (6)	26.1 (6)	47.8 (11)
	No	3.3 (5)	41.4 (63)	55.3 (84)
Χ^2^ = 17.917 df = 2 *p* < 0.01				

*N*, number of participants. %, percentage of participants.

## Data Availability

The original contributions presented in this study are included in the article. Further inquiries can be directed to the corresponding author.
